# Supplementary material for: Genotype and Phenotype Analyses of a Novel WFS1 Variant (c.2512C>T p.(Pro838Ser)) Associated with DFNA6/14/38
Source: Genes (Basel). 2023 Feb 10;14(2):457. doi: 10.3390/genes14020457 (PMC9957259; doi:10.3390/genes14020457)
Supplement: Supplementary file 1 [file genes-14-00457-s001.zip › Figure S1.pdf]

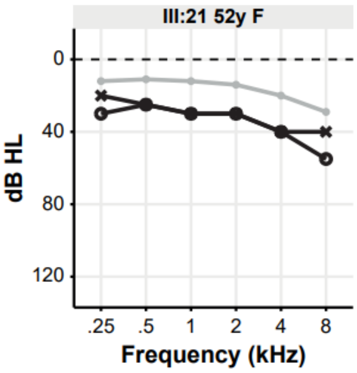

Supplemental Figure S1. Audiogram of subject III:21. The pure tone air conduction thresholds in dB HL of 0.25 to 8 kHz of subject III:21 who was not identified with the c.2512C>T p.(Pro838Ser) variant and has a hearing phenotype not typically for DFNA6/14/38. Black lines with circles represent the right ear, black lines with crosses represent the left ear, grey lines and dots represent the age- and gender-specific 95th percentile. dB HL, decibel hearing level; f, female; kHz, kilo hertz; y, years.
