# Supplementary material for: Genotype and Phenotype Analyses of a Novel WFS1 Variant (c.2512C>T p.(Pro838Ser)) Associated with DFNA6/14/38
Source: Genes (Basel). 2023 Feb 10;14(2):457. doi: 10.3390/genes14020457 (PMC9957259; doi:10.3390/genes14020457)
Supplement: Supplementary file 1 [file genes-14-00457-s001.zip › Figure S2.pdf]

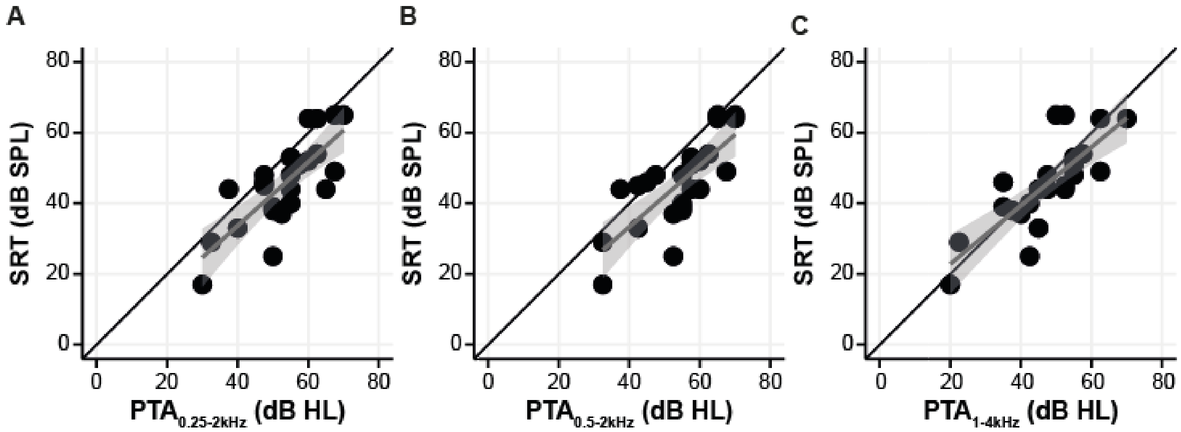

Supplemental Figure S2. SRT as function of PTAs. The SRT in decibel sound pressure level (dB SPL) as function of three combinations of frequencies determining the pure tone average in decibel hearing level (dB HL). **(a)** PTA of 0.25, 0.5, 1 and 2 kHz. **(b)** PTA of 0.5, 1 and 2 kHz (*i.e.* Fletcher index). **(c)** PTA of 1, 2 and 4 kHz. Given the shape of the audiogram, the SRT is best described by the PTA based on 1, 2 and 4 kHz.
