# Supplementary material for: Genotype and Phenotype Analyses of a Novel WFS1 Variant (c.2512C>T p.(Pro838Ser)) Associated with DFNA6/14/38
Source: Genes (Basel). 2023 Feb 10;14(2):457. doi: 10.3390/genes14020457 (PMC9957259; doi:10.3390/genes14020457)
Supplement: Supplementary file 1 [file genes-14-00457-s001.zip › Table S1.pdf]

Supplemental Table S1. Results of questionnaires and ENT consultation in all subjects.

Subject	Age (y)	HL	Age of HL onset[a]	Use of hearing aids	Risk factors for acquired HL	Vestibular symptoms	DHI	Head Impulse Test[b]	Other
III:02	77	Yes	0-10y	No	-	Instability after stroke	38	Normal	-
III:04	72	Yes	0-10y	Yes	-	Dizziness when standing up	NC	Normal	-
III:08	72	No	N/a	N/a	-	-	0	Not performed (arthrosis)	Mild myringosclerosis RL
III:11	70	Yes	Congenital	No	Recurrent ear infections	Vertigo; problems with balance on bicycle	34	Normal	Tinnitus; myringosclerosis R
III:13	69	Yes	11-30y	Yes	-	-	0	Normal	-
III:17	62	No	N/a	N/a	Meningitis (35y)	-	0	Normal	Mild myringosclerosis RL
III:18	65	No	N/a	N/a	Noise exposure	-	0	Normal	-
III:21	52	Yes	31-50y	Yes	Noise exposure	-	NC	Normal	Tinnitus
III:22	59	Yes (unilateral)	0-10y	No	Recurrent ear infections; noise exposure	-	8	Normal	Tinnitus
III:25	65	Yes	0-10y	Yes	-	-	18	Normal	-
III:27	59	Yes	31-50y	Yes	-	-	NC	Normal	Tinnitus; exostosis R; mild myringosclerosis L
IV:03	43	Yes	Congenital	No	Recurrent ear infections	-	NC	Normal	Tinnitus; apparent life-threatening event (infant)
IV:05	49	No	N/a	No	Recurrent ear infections; noise exposure; antibiotic use	-	0	Normal	Myringosclerosis RL
IV:07	49	Yes	0-10y	Yes	-	-	0	Normal	-
IV:09	46	Yes	11-30y	Yes	-	-	0	Normal	Tinnitus
IV:12	42	No	N/a	N/a	-	-	NC	Normal	-
IV:13	30	Yes	Congenital	Yes	Noise exposure	-	14	Normal	Tinnitus; hypothyroidism

V:01	5	Yes	0-10y	Yes	Recurrent ear infections (with ear tubes)	-	NC	Not performed (age)	Passed neonatal hearing screening
V:04	11	Yes	0-10y	Yes	-	Mildly delayed motor development	0	Normal	Passed neonatal hearing screening

[a] Self-reported age of onset. [b] If the head impulse test has not been performed, the reason is indicated in brackets. DHI, dizziness handicap inventory; HL, hearing loss; N/a, not applicable; NC, not completed; R, right ear; RL, right and left; L, left; y, years.
