# Supplementary material for: Genotype and Phenotype Analyses of a Novel WFS1 Variant (c.2512C>T p.(Pro838Ser)) Associated with DFNA6/14/38
Source: Genes (Basel). 2023 Feb 10;14(2):457. doi: 10.3390/genes14020457 (PMC9957259; doi:10.3390/genes14020457)
Supplement: Supplementary file 1 [file genes-14-00457-s001.zip › Table S2.pdf]

Supplemental Table S2. Air conduction thresholds of all DFNA6/14/38 subjects.

Subject	Gender	Age at examination (y)	Source	Hearing thresholds (dB HL) for frequencies (kHz)					
				**0.25**	**0.5**	**1**	**2**	**4**	**8**
III:02	Female	77	Right ear	50	50	50	25	40	45
			Left ear	60	55	50	35	20	45
			Mean	55	52.5	50	30	30	45
			P50	14	16	20	27	37	50
			P95	27	30	35	45	58	74
III:04	Female	72	Right ear	80	70	65	50	30	60
			Left ear	65	65	60	55	50	60
			Mean	72.5	67.5	62.5	52.5	40	60
			P50	11	12	15	21	29	40
			P95	22	24	28	37	49	65
III:11	Female	70	Right ear	75	80	70	60	30	35
			Left ear	80	70	65	60	40	40
			Mean	77.5	75	67.5	60	35	37.5
			P50	10	11	14	19	27	37
			P95	20	22	26	34	47	62
III:13	Female	69	Right ear	55	70	80	70	60	30
			Left ear	55	65	70	65	55	60
			Mean	55	67.5	75	67.5	57.5	45
			P50	9	10	13	18	25	35
			P95	19	21	25	33	44	60
III:25	Female	65	Right ear	65	65	60	50	50	75
			Left ear	55	60	55	55	50	75
			Mean	60	62.5	57.5	52.5	50	75
			P50	7	8	10	14	20	29
			P95	16	18	21	27	37	52
III:27	Female	59	Right ear	65	55	55	30	50	65
			Left ear	55	55	50	40	45	55
			Mean	60	55	52.5	35	47.5	60
			P50	5	6	7	9	14	20
			P95	14	15	16	20	29	40
IV:03	Male	43	Right ear	55	55	60	55	50	25
			Left ear	55	60	60	55	50	25
			Mean	55	57.5	60	55	50	25
			P50	1	1	2	3	6	9
			P95	8	9	10	11	15	21
IV:07	Male	49	Right ear	40	50	55	60	20	0
			Left ear	45	55	55	55	15	5
			Mean	42.5	52.5	55	57.5	17.5	2.5
			P50	2	2	3	6	9	14
			P95	10	11	12	15	20	29
IV:09	Male	46	Right ear	55	55	50	55	35	5
			Left ear	45	50	55	35	35	10
			Mean	50	52.5	52.5	45	35	7.5
			P50	1	2	2	4	7	11
			P95	9	10	10	13	17	24
IV:13	Female	30	Right ear	70	70	70	65	55	0
			Left ear	65	65	65	60	50	10
			Mean	67.5	67.5	67.5	62.5	52.5	5
			P50	0	0	0	0	0	1
			P95	8	8	9	9	9	11
V:01	Female	5	Right ear	65	70	65	35	20	15

			Left ear	65	65	60	40	20	30
			Mean	65	67.5	62.5	37.5	20	22.5
V:05	Female	11	Right ear	35	40	40	25	0	5
			Left ear	45	45	45	20	0	10
			Mean	40	42.5	42.5	22.5	0	7.5

Air conduction thresholds in dB hearing level (dB HL) of 0.25 to 8 kHz and the age- and gender-specific 50th and 95th percentile (ISO 7029:2017 [40]) of all DFNA6/14/38 subjects. dB HL, decibel hearing level; kHz, kilohertz; y, years.
